# Scientific Items

**Published:** 1886-03-20

**Authors:** 


### SCIENTIFIC ITEMS.

**Rattlesnakes.**—In quantity, the venom injected by a large and active rattlesnake is about four minims, or two for each fang. He can strike twice or thrice in rapid succession with deadly effect, but soon the glands are unable to keep up the supply, and he will require some minutes to recuperate. Snake charmers usually sear the glands with a hot iron, leaving the fangs intact, but only capable of making a slight flesh wound. Too much care cannot be exercised in dissecting a rattlesnake's head, for the glands secrete for sometime after death, and a little of the virus goes a long way.

In New Mexico and on the Staked Plains in Texas, where the nights are cool, it is the rattlesnake's sociable custom to crawl between a traveler's blankets and snuggle close to him till morning. Numbers of them are killed in camp every year by soldiers campaigning in that section; but as the rattlesnakes never abuse hospitality by biting the sleeper, few accidents happen. Still, there are men who, when out on a hard march, prefer to sleep alone.—*Scientific American.*

**Wonders of the Sea.**—The sea occupies three-fifths of the surface of the earth. At the depth of about 3,500 feet, waves are not felt. The temperature is the same, varying only a trifle from the ice of the pole to the burning sun of the equator. A mile down, the water has a pressure of over a ton to the square inch. If a box six feet deep were filled with sea water and allowed to evaporate under the sun, there would be two inches of salt left on the bottom. Taking the average depth of the ocean to be three miles, there would be a layer of pure salt 230 feet thick on the bed of the Atlantic. The water is colder at the bottom than at the surface. In the many bays on the coast of Norway, the water often freezes at the bottom before it does above.

Waves are very deceptive. To look at them in a storm, one would think the water traveled. The water stays in the same place, but the motion goes on. Sometimes in storms these waves are forty feet high, and travel fifty miles an hour—more than twice as fast as the swiftest steamer. The distance from valley to valley is generally fifteen times the height, hence a wave five feet high will extend over seventy-five feet of water. The force of the sea dashing on Bell Rock is said to be seventeen tons for each square yard. Evaporation is a wonderful power in drawing water from the sea. Every year a layer of the entire sea fourteen feet is taken up into the clouds. The winds bear their burden into the land, and the water comes down in rain upon the fields, to flow back at last through rivers. The depth of the sea presents an interesting problem. If the Atlantic were lowered 6,564 feet, the distance from shore to shore would be half as great, or 1,500 miles. If lowered a little more than three miles, say 19,680 feet, there would be a road of dry land from Newfoundland to Ireland. This is the plain on which the great Atlantic cables were laid. The Mediterranean is comparatively shallow. A drying up of 660 feet would leave three different seas, and Africa would be joined with Italy. The British Channel is more like a pond, which accounts for its choppy waves.

It has been found difficult to get correct soundings of the Atlantic. A midshipman of the navy overcame the difficulty, and a shot weighing thirty pounds carries down the line. A hole is bored through the sinker, through which a rod of iron is passed, moving easily back and forth. In the end of the bar a cup is dug out, and the inside coated with lard. The bar is made fast to the line, and a sling holds the shot on. When the bar which extends below the ball, touches the earth, the sling unhooks and the shot slides off. The lard in the end of the bar holds some of thes and or whatever may be on the bottom, and a drop shuts over the cup to keep the water from washing the sand out. When the ground is reached, a shock is felt as if an electric current had passed through the line.—*Electrical Review.*

**The Cobra Poison.**—Dr. R. Morris Wolfenden, in a communication to the Royal Society, reported he had found the poisonous properties of the cobra (the East Indian hooded snake, Naga tripudians) poison to reside entirely in the proteids—globulin, serum albumen and syntonin. The formerly reputed power of the potassium permanganate, as an antidote for snake bites, is, says Nature, explained by its action upon albumens, which it converts into oxy-protosulphonic and other acids; but as it oxidizes all albumens indifferently, it can only be of service locally when immediately injected into the spot bitten.

It may be noted in this connection that Surgeon-Major Roy, in a communication to the Lancet, suggests that snake poison will be found to neutralize the poison of hydrophobia, and offers to supply a mixture of cobra poison and glycerin for injection. It has struck him "that nature could not be so improvident and callous as to leave us thoroughly helpless and unprotected." The question is has hydrophobia ever been cured by the proposed remedy?—*Druggists Circular and Chemical Gazette.*

**Cleaning Hands After Laboratory Work.**—To clean the hands after work in the laboratory or shop, rub with a little petroleum jelly and wash with warm water.—*Ibid.*

**An Egg Register** has been patented by Mr. Casper Marti, of Minneapolis, Minn. A suitable box has a pivoted platform, with apertures at one end for receiving eggs, a counting device being operated from the platform, whereby the eggs will be automatically counted and the number registered.—*Scientific American.*

A machine for scraping rattan has been patented by Mr. James M. Devanery, of Hoboken, N. J.—*Ibid.*

